# From Three-Months to Five-Years: Sustaining Long-Term Benefits of Endovascular Therapy for Ischemic Stroke

**DOI:** 10.3389/fneur.2021.713738

**Published:** 2021-07-26

**Authors:** Aravind Ganesh, Johanna Maria Ospel, Martha Marko, Wim H. van Zwam, Yvo B. W. E. M. Roos, Charles B. L. M. Majoie, Mayank Goyal

**Affiliations:** ^1^Department of Clinical Neurosciences, University of Calgary, Calgary, AB, Canada; ^2^Department of Neuroradiology, University Hospital Basel, Basel, Switzerland; ^3^Department of Neurology, Medical University of Vienna, Vienna, Austria; ^4^Department of Radiology, Maastricht University Medical Centre, Maastricht, Netherlands; ^5^Department of Radiology, Amsterdam UMC, Amsterdam, Netherlands; ^6^Department of Neurology, Amsterdam UMC, Amsterdam, Netherlands; ^7^Department of Radiology, University of Calgary, Calgary, AB, Canada; ^8^Hotchkiss Brain Institute, University of Calgary, Calgary, AB, Canada

**Keywords:** cerebrovascular disease, ischemic stroke, endovascular treatment, long-term outcome, post-acute care

## Abstract

**Background and Purpose:** During the months and years post-stroke, treatment benefits from endovascular therapy (EVT) may be magnified by disability-related differences in morbidity/mortality or may be eroded by recurrent strokes and non-stroke-related disability/mortality. Understanding the extent to which EVT benefits may be sustained at 5 years, and the factors influencing this outcome, may help us better promote the sustenance of EVT benefits until 5 years post-stroke and beyond.

**Methods:** In this review, undertaken 5 years after EVT became the standard of care, we searched PubMed and EMBASE to examine the current state of the literature on 5-year post-stroke outcomes, with particular attention to modifiable factors that influence outcomes between 3 months and 5 years post-EVT.

**Results:** Prospective cohorts and follow-up data from EVT trials indicate that 3-month EVT benefits will likely translate into lower 5-year disability, mortality, institutionalization, and care costs and higher quality of life. However, these group-level data by no means guarantee maintenance of 3-month benefits for individual patients. We identify factors and associated “action items” for stroke teams/systems at three specific levels (medical care, individual psychosocioeconomic, and larger societal/environmental levels) that influence the long-term EVT outcome of a patient. Medical action items include optimizing stroke rehabilitation, clinical follow-up, secondary stroke prevention, infection prevention/control, and post-stroke depression care. Psychosocioeconomic aspects include addressing access to primary care, specialist clinics, and rehabilitation; affordability of healthy lifestyle choices and preventative therapies; and optimization of family/social support and return-to-work options. High-level societal efforts include improving accessibility of public/private spaces and transportation, empowering/engaging persons with disability in society, and investing in treatments/technologies to mitigate consequences of post-stroke disability.

**Conclusions:** In the longtime horizon from 3 months to 5 years, several factors in the medical and societal spheres could negate EVT benefits. However, many factors can be leveraged to preserve or magnify treatment benefits, with opportunities to share responsibility with widening circles of care around the patient.

## Introduction

**Figure 1:**
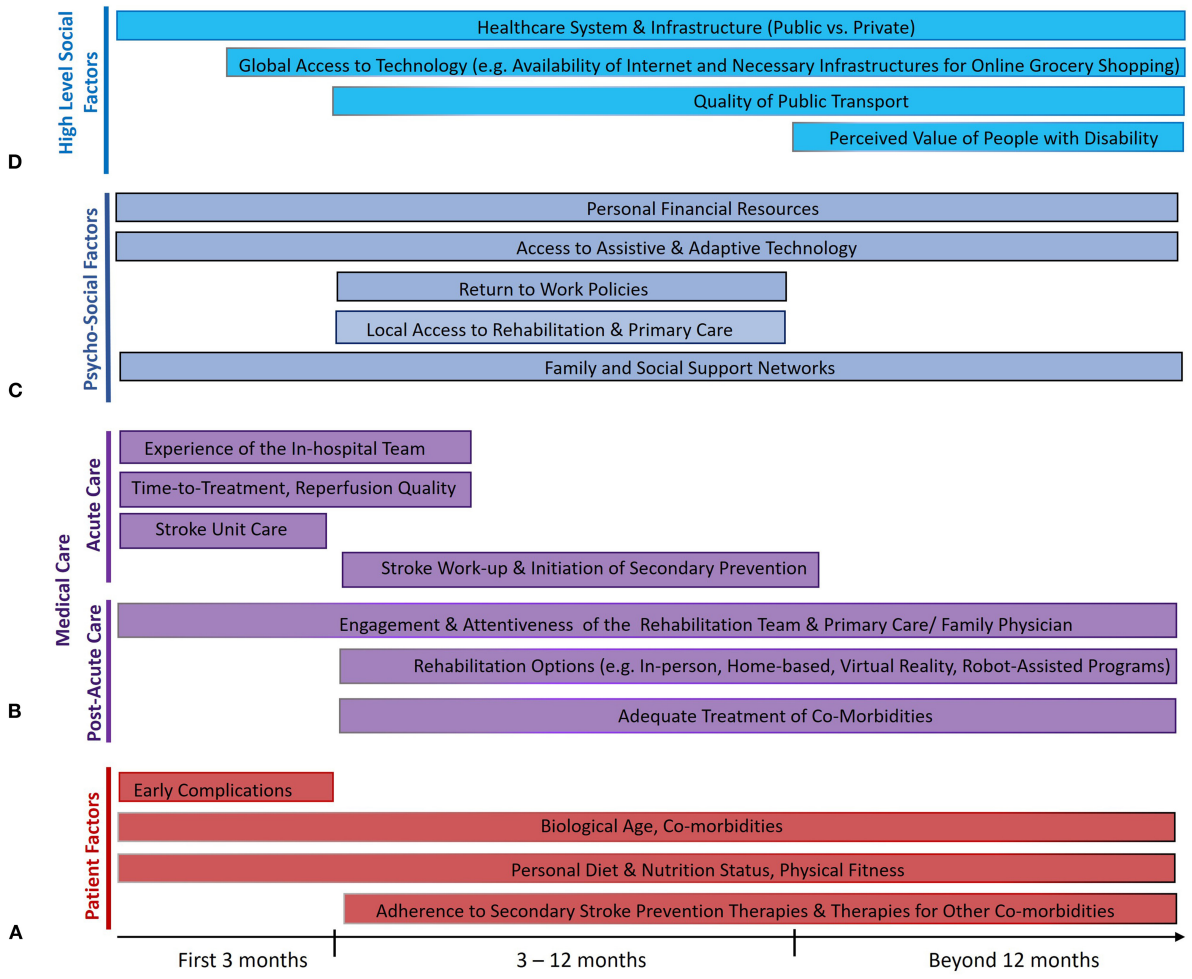
Factors that influence the maintenance of treatment benefits of endovascular therapy (EVT) from 3 months to 5 years after ischemic stroke from large vessel occlusion. Several factors relate to the individual patients themselves **(A)**, some of which are modifiable (like risk of early complications) and rely on effective collaboration between patients and physicians, while several others directly relate to the quality of acute and post-acute medical care **(B)**. Many psychosocioeconomic factors in the life of the patient also play a crucial role **(C)**, as do higher-level societal factors **(D)** that influence the ability of the patient to reintegrate post-stroke and live a fulfilling life. The length of the bars reflects the portion of the post-stroke time period where each factor is thought to play an important role.

Endovascular therapy (EVT) is a highly efficacious treatment for acute ischemic stroke with large vessel occlusion (LVO), promoting post-stroke functional independence (1). Through successful reperfusion of brain tissue, EVT results in lower post-treatment infarct volumes when performed rapidly (2–4). However, fast and successful EVT alone does not guarantee a good outcome. Several critical factors operate in the post-stroke period that can influence the 3-month recovery of the patient. Some are unmodifiable, like advanced age and comorbidities like cardiovascular disease or cancer (Figure 1A) (5). Others are modifiable through attention to the quality of post-acute care, such as the occurrence of post-stroke complications like pneumonia or deep vein thrombosis (5, 6).

Notwithstanding the various potential pitfalls in stroke recovery from EVT to 3 months, the longer time horizon from 3 months to 5 years is fraught with even greater uncertainty. Some patients may experience further recovery from disability beyond 3 months, while some others successfully maintain their independence, with magnification of treatment-related differences in terms of long-term disability and mortality (7). On the other hand, EVT-related benefits may be eroded by recurrent strokes, accrual of non-stroke-related disability, or by non-stroke-related deaths, especially since stroke occurs more often in elderly people with progressive comorbidities. This raises the question of how we may maximally sustain the initial gains from EVT.

In this review, 5 years after EVT became the standard of care for acute ischemic stroke with LVO, we examine the current state of the literature on 5-year post-stroke outcomes, with particular attention to the modifiable factors that influence the evolution of outcome of the patients between 3 months and 5 years post-stroke. This knowledge may help us better ensure that the therapeutic benefits of EVT are sustained to the greatest possible extent until 5 years post-stroke and beyond.

## Literature Search

We searched the literature for studies that (1) involved patients with ischemic stroke and (2) examined a post-stroke outcome of interest beyond the 3-month period (search strategy in the Supplementary Material). Although we were most interested in studies that examined long-term outcomes after EVT or LVO-associated stroke, there continues to be a paucity of high-quality studies examining longer-term outcomes in this specific population. Therefore, we took a more inclusive approach and considered studies in the general ischemic stroke population as well, since most aspects of post-acute care are not unique to the LVO population. We limited the search to studies of humans published in English. The literature search is up-to-date as of April 30, 2021. We excluded case reports, case series, and opinions or editorials.

### Maximizing Three-Month Outcomes With EVT

The sustenance of EVT benefits between 3 months and 5 years post-stroke is predicated on maximizing 3-month treatment effects in the first place. Therefore, it is worth briefly reviewing key factors of acute stroke care that can optimize 3-month EVT benefits (Figure 1B).

Tremendous gains have been made in EVT technology, techniques, and workflow. Improvements in thrombectomy devices (specifically stent retrievers) were crucial to the dawn of successful EVT for stroke (8, 9), and the continued evolution of these devices—with better size choices and longer, more radio-opaque designs—and of EVT training programs holds promise for further enhancing EVT benefits (10–12). Speed is also critical (13); indeed, shortened treatment times from IMS-III (Interventional Management of Stroke trial-III) to the major EVT trials in 2015 helped drive efficacy (14, 15). Further refinement of EVT techniques like CAPTIVE (continuous aspiration prior to intracranial vascular embolectomy) (16) or BADDASS (BAlloon guiDe with large bore DISTAL ACCESS catheter with dual aspiration with Stent retriever as Standard approach) (17) is also crucial to continue improving reperfusion rates. The population benefitting from EVT continues to expand, such as “late-window” patients with salvageable penumbra (18, 19) and potentially patients with more extensive early ischemic changes for whom trials are ongoing (20, 21). Three-month outcomes may be further improved by neuroprotective therapies (22); a promising treatment is nerinetide, which may improve outcomes in patients not receiving alteplase (23). Artificial intelligence and machine learning approaches may further improve outcomes through decision support for stroke identification/triage, imaging interpretation, and patient selection for treatment (24, 25).

Following EVT, attention to post-acute care, ideally on organized stroke units, is essential to prevent or mitigate complications like aspiration pneumonia or deep vein thrombosis, which can rapidly negate EVT benefits (5, 6). Larger, systems-level factors also matter, such as whether care occurs in the context of integrated systems of stroke care. Such systems involve concerted efforts across the continuum from prehospital care all the way to post-stroke rehabilitation and secondary prevention (26) and may promote lower 30-day mortality (27). Even in regions with more fragmented systems, the adoption of certain concerted approaches to stroke workflow, such as prehospital notification of incoming “code strokes” and rapid patient triage, stroke team activation, and neuroimaging completion, can improve onset-to-groin-puncture times and thus improve 3-month outcomes (28, 29). The organization of stroke systems in the field to ensure efficient transport of patients with LVO for EVT remains a work in progress (30). Several scales have been developed for prehospital identification of LVO, with attendant limitations (31, 32), and geographical modeling of optimal transport options has emerged as an important technology to guide routing decisions (33, 34).

### Relationship Between Three-Month and Five-Year Post-stroke Outcomes

The enduring burden of long-term disability in ischemic stroke has been reported in many cohorts, with about 31–36% of patients being functionally disabled patients 5 years post-stroke (35–38). Three-month modified Rankin Scale (mRS) scores strongly predicted 5-year disability in the population-based Oxford Vascular Study (OXVASC), implying that treatments like EVT that reduce 3-month disability likely promote long-term functional independence (39).

As for mortality, at 1 year post-stroke and beyond, the most frequent causes are respiratory infections and cardiovascular disease (40). Functional dependency, with attendant issues of immobility and incontinence, is associated with complications like infections and pressure sores (41). Observational studies have shown that early post-stroke disability predicts long-term mortality (key studies shown in Table 1). These data suggest that early disability reductions from EVT will likely translate into lower long-term mortality, without much erosion by non-stroke-related deaths. Cognitive impairment is a well-recognized post-stroke complication, progressing to dementia in up to one-third of patients (48, 49), with dementia incidence being nearly 50 times higher 1 year post-stroke compared with the general population (50). Post-stroke dementia contributes to dependency (51, 52), institutionalization, and mortality (53). In OXVASC, each 3-month mRS increment was associated with higher 5-year risk of dementia (54).

**Table 1:** Key observational studies examining the relationship of short-term post-stroke disability or functional outcome to 5-year (or longer-term) mortality.

**Study**	**Main finding**
Perth Community Stroke Study (42)	Post-stroke disability on the Barthel Index (<20/20 at around 1 month), particularly urinary incontinence, significantly predicted 5-year death
Prospective admissions-based study at the University of Rome (43)	Only included minor strokes (30-day mRS ≤ 2) and it was found that mRS = 2 was associated with a hazard ratio (HR) of 3.4 for 10-year mortality
Rochester-based retrospective medical record review (40)	On reviewing cause-specific mortality over 10 years or more after first ischemic stroke, it was found that mRS of 4 or 5 was associated with higher mortality.
Riksstroke study (Sweden) (44)	Reported HRs of 1.7, 2.5, and 3.8, respectively, for 3-year mortality for 3-month mRS of 3, 4, and 5 compared with 0–2
Athens Stroke Registry (45)	Patients with worse 3-month mRS scores had higher mortality, after adjusting for comorbid risk factors (relative mortality risk increases of 18, 55, 80, 157, and 472%, respectively, for mRS 1 through 5 vs. 0).
Oxfordshire Community Stroke Project, Lothian Stroke Registery, and the First International Stroke Trial (combined analysis) (46)	Among 7,710 ischemic stroke patients registered between 1981 and 2000 and followed for up to 19 years, functional dependence was a significant predictor of mortality in each cohort, with the median survival being 9.7 years in independent patients and 6.0 years in dependent patients.
Oxford Vascular Study (OXVASC)	Detailed analysis of cause-specific mortality among 1,606 patients found that 54.8% of deaths after 3 months attributable to stroke-related complications occurred after 1 year (39), with each increment of 3-month mRS being associated with higher 5-year mortality (47).

*mRS, modified Rankin Scale*.

Healthcare costs also reflect long-term treatment effects. Three-month functional outcome again predicts long-term post-stroke costs. A systematic review of costing studies between 2004 and 2015 found that costs consistently increased with increasing mRS (55). In OXVASC, each increment of worsening 3-month mRS was associated with higher 5-year healthcare/social care costs (47), regardless of premorbid disability (56). Analyses of the North-East Melbourne Stroke Incidence Study (NEMESIS) have shown that 5-year outcomes provide a robust estimate of lifetime post-stroke costs (57). Long-term costs are closely tied to institutionalization, i.e., admission to residential care or nursing homes, affecting 9–15% of patients by 5 years post-stroke (36, 37, 58–60) and over 40% of initially hospitalized patients with severe strokes (61–63). Unsurprisingly, early disability predicts 5-year institutionalization. The Erlangen Stroke Project found that urinary incontinence on the Barthel Index at 7 days conferred a four-fold higher risk of 12-month institutionalization (64). In OXVASC, 1-month/3-month mRS predicted 5-year institutionalization (>35% with mRS of 3–5 vs. <10% with mRS 0–2) (36, 54).

Higher post-stroke disability is also associated with poorer quality of life. Indeed, the 3-year Australian POISE (Psychosocial Outcomes In Stroke) study found that functional independence at 28 days was the strongest predictor of return-to-work within 1 year post-stroke (65). In OXVASC, each 3-month mRS increment was associated with worse quality-of-life ratings and 5-year quality-adjusted life expectancy (QALE) (54, 66). The VISTA (Virtual International Stroke Trials Archive) collaborators found that 3-month mRS scores accounted for 65–71% of variation in health utilities generated using EQ-5D data for different countries (67).

From these observational data, we may extrapolate that 3-month EVT benefits will likely translate into lower 5-year disability, mortality, institutionalization, and care costs and higher quality of life/QALE. This suggests that the 3-month benefits are probably preserved and potentially magnified at 5 years, but a caveat is that many/most of the patients in these cohorts did not have LVOs (although OXVASC reported sensitivity analyses in potentially “treatable” strokes) (39). Preliminary real-world data showing these long-term benefits have come from the analyses of the MR CLEAN (Multicenter Randomized Clinical trial of Endovascular treatment for Acute ischemic stroke in the Netherlands) and REVASCAT (a randomized trial of revascularization with SOLITAIRE FR® device vs. best medical therapy in the treatment of acute stroke due to anterior circulation LVO presenting within 8 h of symptom onset) trials. An analysis of 2-year mRS data from 391 of 500 patients enrolled in MR CLEAN (78.2%) showed an adjusted OR of 1.68 (95% CI 1.15–2.45) for a shift of mRS in favor of EVT (68). One-year mRS data from REVASCAT, available for 205 of 206 patients (99%), showed that 89% of the positive treatment effect was already observed at 90 days (69). In REVASCAT, EVT was also associated with better cognitive performance at 3 months and 1 year on the trail-making—test part B, especially among patients with mRS 0–2 (70).

Interestingly, a recent OXVASC analysis that applied prognostic weights derived for each level of the 3-month mRS to EVT trial data estimated very similar long-term treatment effects as the actual MR CLEAN and REVASCAT analyses. For example, OXVASC estimated a 2.5% lower mortality (95% CI −7.1 to 12.0%) and 0.06 additional QALY (0.003–0.13) in the REVASCAT EVT arm at 1 year, similar to the non-significant 1% mortality difference and 0.12 (0.03–0.22) utility difference reported in the 1-year REVASCAT analysis (69). Similarly, OXVASC estimated a 5.5% lower mortality (−0.5 to 11.4%) and 0.14 additional QALY (0.06–0.23) in the MR CLEAN EVT arm at 2 years, which was close to the 5% mortality and 0.10 (0.03–0.16) utility differences reported in the 2-year MR CLEAN analysis (68). Buoyed by these robust estimates, the 5-year benefits of EVT were extrapolated using weighted ordinal analyses of pooled 3-month mRS results of all major EVT trials. Endovascular therapy was predicted to confer an 11% lower risk (95% CI 9–14%) of death/dementia/institutionalization, a $10,193 (7,405–12,981) reduction in healthcare/social care costs, and an additional 0.55 (0.43–0.66) QALYs over 5 years vs. control treatments. A subsequent analysis from the HERMES collaboration estimated that every 10 min of earlier EVT results in an average gain of 39 days of disability-free life and increases net monetary benefit by $10,519 for healthcare costs and $10,915 for societal costs over the lifetime of a patient, indicating the long-term benefits of faster EVT (71).

### Medical Care-Related Factors Influencing Five-Year Stroke Recovery and Outcomes

Importantly, the strong group-level observational and clinical trial data for the extrapolation of 3-month benefits of EVT to 5 years and beyond by no means guarantee the maintenance of 3-month benefits for individual patients. At the individual level, there are numerous factors occurring as part of the medical care of the patient (both physician- and patient-dependent) that likely influence how the long-term EVT outcome of the patient will play out (Figure 1B).

Firstly, the 3-month disability need not guarantee 5-year disability. Whereas, post-stroke recovery was conventionally thought to occur mostly in the first 3 months post-stroke (72), rehabilitation strategies like constraint-induced movement therapy (CIMT) have been shown to be effective in the 3- to 9-month window (73, 74), indicating that patients may demonstrate late functional improvement (75). In OXVASC (76) and in an analysis of three randomized multicenter trials of acute ischemic stroke (2,555 patients), such improvement (by ≥1 mRS grades) was observed in about one in four patients with ischemic stroke between 3 and 12 months post-stroke (77). Whereas, analyses of 11 rehabilitation pilot studies demonstrated a gradient of recovery fading to asymptotic levels after about 18 months post-stroke (78), functional improvement was also seen in about 1 in 10 patients in OXVASC between 1 and 5 years post-stroke (76). Although such late improvements, particular between 3 and 12 months, seem more common among those with lacunar strokes (76), patients who demonstrated late improvement in OXVASC, regardless of subtype, had lower 5-year mortality, institutionalization, and healthcare/social care costs (79). These findings should motivate clinicians and patients to maximize late recovery in practice. Robot-assisted rehabilitation holds promise for promoting intensive, interactive, and individualized practice, but methodologically limited studies to date have only shown small effects on motor control and medium effects on strength (80). Augmentation of rehabilitation interventions with virtual reality, particularly involving a gaming component, improves treatment gains by over 10% compared with conventional approaches (81). These approaches may help further promote late recovery in the future.

In addition, attention to secondary stroke prevention and care for post-stroke complications is critical. It is essential to address and control all modifiable cardiovascular risk factors to prevent recurrent stroke. Anticoagulation for atrial fibrillation is one important example, given the high stroke recurrence in the absence of anticoagulation. Some observational studies suggest that early initiation of direct oral anticoagulants post-stroke may be associated with an acceptably low risk of ICH (82, 83); randomized controlled trials are currently investigating the optimal time point to start anticoagulation (e.g., ELAN—NCT03148457, OPTIMAS—NCT03759938, TIMING—NCT02961348) (84). Organized clinical follow-up is associated with lower hospitalization rates several months post-stroke (85–88). There is a wide variation in the availability of secondary prevention services and medical follow-up (89, 90). In a recent American study, 59.3% of patients had primary care follow-up within 1 month post-stroke and only 24% had neurology/stroke service follow-up (87). Similar challenges have been noted in other countries; in Sweden, only 75% of patients in the Riksstroke registry had 90-day follow-up (91). The added benefits of predefined care models and specialized stroke prevention clinics are being systematically studied in clinical trials (92, 93), which may facilitate their wider adoption.

Moreover, patient compliance and lifestyle modification are critical to maintain functional independence. Beyond prescriptions, patients need appropriately tailored information and education to mitigate risk and promote timely recognition of recurrent strokes (94). The quality of patient/caregiver educational strategies is quite variable, with some approaches showing limited effect on long-term outcomes (95, 96). Patients also benefit from organizational and behavioral interventions to meet secondary prevention goals like blood pressure or low-density lipoprotein targets (97, 98). Underscoring the importance of follow-up, patients without 90-day follow-up have lower medication compliance (91). Only around 65% of patients adhere to statins (99, 100), while 60% adhere to anticoagulation (101–103). Barriers to adherence include challenges with self-care, limited knowledge about stroke and its dangers, frequent medication changes, and high treatment burden and complexity (104). Lifestyle modification, especially smoking cessation, is key for secondary prevention. Yet in a recent analysis of the National Health and Nutrition Examination Survey and the Behavioral Risk Factor Surveillance System survey, active smoking had not become less prevalent among stroke survivors over the past 20 years in the United States, in contrast to the general population (105).

Aside from recurrent cardiovascular events, infections are an important cause for readmission post-stroke (106). Such infections, including aspiration pneumonia (39), are associated with increased mortality (107). Particular vigilance is required for patients with dysphagia, associated with pneumonia and increased morbidity/mortality (108). Importantly, swallowing therapy improves long-term survival (109), emphasizing the importance of multidisciplinary care, in this case including speech and language pathologists.

Furthermore, depression affects one-third of patients after stroke and adversely affects long-term outcomes. Optimal treatment options and benefit of antidepressants for daily activities remain uncertain, but early recognition with a combination of pharmacological and non-pharmacological approaches is prudent (110). Recent trials of fluoxetine in the early post-stroke period have shown benefits for mood and emotional control (111, 112) with reduced incidence of new post-stroke depression (112), but no benefits for functional outcomes.

Based on these insights, we can identify a set of “action items” for stroke teams to address between 3 months and 5 years, ideally in tandem with primary care and multidisciplinary teams, to help maximize long-term EVT benefits (Table 2).

**Table 2:** Medical action items for stroke teams to address between 3 months and 5 years post-stroke to ensure maintenance of EVT benefits.

**Aspect of stroke care**	**Goal or action**
Stroke rehab	•Educate patients/payers about potential for late functional recovery •Promote multidisciplinary rehab through early supported discharge (113) •Maximize late functional improvement beyond 3 months using proven strategies (like constraint-induced movement therapy) •Promote regular exercise/mobilization and/or positional changes to mitigate long-term complications of immobility
Clinical follow-up	•Provide organized clinical follow-up for stroke survivors at 3 months and beyond through dedicated stroke prevention clinics (86) •Use multiple types of communication and reminders and liaise with primary care physicians to minimize patients that are “lost to clinical follow-up”
Secondary stroke prevention	•Ensure that underlying mechanisms and risk factors have been appropriately investigated and treated using a multidisciplinary approach as needed •Provide education to patients about stroke (to ensure prompt recognition and treatment of future events) and importance of risk factor management •Support lifestyle modification including smoking cessation and attainment of blood pressure and lipid targets using behavioral interventions and longer-term telephone-based follow-ups (114) •Support self-care (e.g., with rehab specialists) and minimize the complexity of medication regimens to promote adherence (104) •Dedicated quality improvement interventions with pharmacists (115) and smartphone-based strategies may help improve medication adherence in certain populations (116, 117)
Infection prevention and control	•Optimize dietary modifications and swallowing therapy/precautions in patients with dysphagia, in concert with speech and language pathologists •Educate patient and caregivers about prompt recognition and treatment of infections •Implement plans for maintenance of hygiene in patients with incontinence, including scheduled changing of indwelling catheters or teaching clean intermittent catheterization if necessary/appropriate
Post-stroke depression	•Educate patient and caregivers about this common complication and monitor for this in follow-up •Prompt identification and use of pharmacological and non-pharmacological management, with input from psychologists and/or psychiatrists as appropriate

### Psycho-Social Factors Influencing Long-Term Stroke Recovery and Outcomes

Aside from psychological effects of the stroke itself, there are several relevant psycho-social factors operating in the immediate environment of the patient that play a major role in their long-term recovery and, thus, their long-term EVT benefit (Figure 1C).

There is a consistent association of lower socioeconomic status and lower education with higher long-term morbidity/mortality post-stroke (118–120). The growing wealth and income disparity worldwide can be expected to contribute to greater disparities in stroke prevention and outcomes (121, 122). Whereas, socioeconomic or insurance status has been studied mostly in relation to acute/in-hospital care in the United States (123, 124), important data on longer-term care metrics have recently come from European studies. Socioeconomic deprivation was associated with lower survival and greater enduring functional impairment on the Barthel Index at 7 years post-stroke in England (125, 126). Higher education was associated with better motor and functional recovery during rehabilitation in Europe (127) and lower 1-year stroke-related mortality in Finland (128), whereas low income was associated with lower 6-month motor improvement post-stroke in Europe (127) and higher 1-year stroke-related mortality in Finland (128). Some of these differences relate to disparities in accessing good-quality care. For example, patients with socioeconomic deprivation were less likely to receive appropriate post-stroke care during 5 years of follow-up in London, including swallowing assessments, medications for atrial fibrillation, and in Black patients, physiotherapy and occupational therapy (129).

In addition, once patients are discharged from the hospital, their access to rehabilitation programs is highly variable. Insurance policies in countries like the United States often restrict stroke patients from accessing rehabilitation after discharge (75). Even in countries with universal healthcare insurance like the United Kingdom and Canada, patients struggle to access rehabilitation services beyond the first few months post-stroke (130). The aforementioned benefits of late post-stroke recovery should incentivize payers to expand coverage for proven late therapies like CIMT (73) beyond 3 months post-stroke, as such investment can pay off with sustained independence and lower healthcare/social care costs.

However, even with excellent post-acute stroke care, patients may suffer from suboptimal management of non-stroke-related comorbidities due to poor access to primary care or allied health professionals. Timely involvement of primary care physicians is enshrined in guidelines for post-acute care (131), yet options may be limited for patients living in remote/rural areas. Financial barriers also hamper secondary prevention efforts in more subtle ways. Besides making healthy eating habits unaffordable, they create competing priorities for patients trying to attend appointments; for example, patients may struggle with the double hit of lost income on the day of an appointment and transport/parking costs (130).

Besides, there is a substantial need for family support post-stroke to optimize physical recovery and outcomes (132–134). The experience of a patient of residual disability post-EVT can be dramatically different depending on how invested their families are in helping them thrive at home. Closely tied to this is the social support network of the patient; besides having a more enriching quality of life, patients with more open and vibrant social networks extending beyond their family are also more likely to be brought in for timely medical attention with future emergencies like stroke (135, 136). Social support also influences more intimate aspects of daily life; a poor relationship with the person feeding them (strangers vs. family/friends) can, for example, worsen meal-skipping, malnutrition, frailty, and isolation among stroke survivors (137). Compounded by changing family and social dynamics, social isolation is a major public health issue (138) and results in worse post-stroke outcomes (139, 140).

Access to assistive or adaptive technology is another huge determinant of whether post-stroke impairments cause functional disability. Robots and other technologies designed to compensate for impaired skills may help patients retain functional independence (141). Technological options also influence post-stroke return-to-work, a major component of self-perceived autonomy (142). Only two-thirds of “working-age” patients achieve return-to-work within 4 years of stroke (143, 144), with contributory factors falling into personal (impairment, adaptation, motivation), rehabilitative (availability, appropriateness), and workplace (demands, support, disability management) domains (145). Relatively simple professional supports may help facilitate return-to-work, like practice typing for office jobs (146). Unsurprisingly, socioeconomic disparities again play a role, with patients who worked in higher management positions more likely to return than blue-collar workers or farmers (147).

These psychosocioeconomic factors extend beyond the typically demarcated circle of care of stroke teams, but there are still important action items to consider (Table 3). Rather than relying on medical expertise, addressing these challenges requires stroke teams to build collaborations with the family of the patient, social networks, and allied-health community partners and to be effective advocates for patients. One powerful way for stroke teams to help attain these goals is by advocating for and joining integrated stroke systems that empower concerted efforts across the continuum of stroke care (26).

**Table 3:** Psychosocioeconomic action items for stroke teams to address or advocate for between 3 months and 5 years post-stroke to help maintain EVT benefits.

**Issue in patient environment**	**Goal or action**
Access to or adequacy of primary care	•Aim for more seamless communication between hospital and primary care physicians (148) •Try to connect patients with primary care physicians in the community who are accepting new patients as part of discharge planning
Access to or ability to attend specialist clinics and rehabilitation sessions	•Be part of telestroke networks to bridge gaps in access to care, and leverage such opportunities to reduce travel burden for patients and families (149, 150) •Advocate for patients to be covered for rehabilitation by payers, e.g., by focusing on potential cost savings of late functional improvement
Knowledge of and ability to afford healthy lifestyle choices and secondary prevention therapies	•Work in concert with social workers to ensure that patients with financial barriers are best connected to available compassionate or subsidized resources and supplementary income (151) •Develop high-quality educational material that does not assume prior knowledge and can be well-understood by patients of different levels of educational backgrounds
Family support and social networks	•Connect patients with limited support networks with community programs or other social support interventions (152) •Actively seek input from rehab team for homecare, home safety, and best affordable technological supports
Return to work options	•Have a member of the multidisciplinary team (e.g., therapists, social worker) be the point person of the patient to advocate for phased transition into the workplace •Advocate for any return-to-work modifications and assistive technologies to maximize success (153, 154)

### High-Level Societal Factors Influencing Long-Term Stroke Recovery and Outcomes

The long-term post-stroke trajectory of a patient is also influenced by much higher-level, upstream societal environmental factors (155). The relevance of such factors is apparent when considering macrogeographical disparities in stroke outcome, evidenced by higher stroke mortality in lower/middle-income countries (LMICs) (120), and microgeographical disparities, evidenced by higher 1-year mortality in disadvantaged parts of a given city (156). Whereas, the general organization of a healthcare system may dictate the access of a patient to care as discussed above, it is worth noting the factors outside the healthcare sphere that influence patient re-engagement post-stroke and, thus, their long-term outcomes (Figure 1D).

How a society organizes its public and private spaces can greatly affect the ability of a patient to have a fulfilling life post-stroke. Physical barriers like inaccessible entryways, bathrooms, and door thresholds can lock even mildly disabled patients out of economic and leisurely pursuits (157). In societies where having a car becomes essential, patients who are unable to drive and rely on specialized transport services have a worse quality of life (158). The availability of accessible and affordable public transport may help mitigate these challenges.

In addition, how a society values people with disability in the workplace and the public sphere may determine successful re-engagement post-stroke. Is there a supportive niche for stroke survivors or are they discriminated against? These attitudes also influence the self-perceptions and ability of the patient to thrive post-stroke. For example, negative attitudes of employers and colleagues (reflecting prevailing societal attitudes) hamper return-to-work post-stroke (159). The experiences of patients of negative public attitudes toward their need for assistance or accommodations can be especially detrimental to their progress (157).

These high-level factors are clearly beyond the control of an individual physician or stroke team. However, the potential impact of addressing such factors through collaborative efforts (Table 4) between policymakers, governments, or private/public partnerships is substantial. In a world of competing demands on resources, this calls for stroke systems to identify and promote highly motivated and visionary health professionals to leadership positions in public and political spheres where they may champion these areas of reform.

**Table 4:** Action items for societies to address to help maintain EVT benefits in stroke survivors at 5 years and beyond.

**Societal domain**	**Goal or action**
Accessible public/private spaces	•Provide ramps/railings, minimizing doorway barriers, etc., at all major buildings or businesses
Accessible and affordable transport options	•Incorporate pathways for safe return to driving, such as formal driving assessment and retraining (160) •Equip public transport vehicles with grips and bar handles on both sides, or provide complimentary walkers, canes, or wheelchairs (157) •Provide discounts for patients with disability for taxi rides (if poor transit options) or bus/train passes
Empowering contributions and engagement of persons with disability in society	•Promote representation and accommodation of persons with disability in the workplace and in decision-making positions •Make public spaces inviting for such individuals through clear signage and symbols •Promote openness of institutions like libraries or theaters to help patients plan and enjoy visits (157)
Development of treatments and technologies to mitigate consequences of disability	•Invest in research and development of adaptive technology and long-term restorative therapies •Invest in the widespread adoption of efficacious technology and treatments

## Discussion

Endovascular therapy is one of the most efficacious therapies in modern medicine. Current evidence from 2-year follow-up of EVT trials and 5-year follow-up from longitudinal studies of ischemic stroke indicates that the 3-month group-level benefits of EVT will likely be sustained at 5 years, further supporting its long-term cost-effectiveness. In this paper, we have examined the various factors that can potentially modify the long-term outcomes of patients after ischemic stroke, drawing on the best available evidence in the literature. The adoption of regular audits and feedback as quality improvement strategies could help healthcare systems optimize these various aspects of patient care and follow-up across the continuum of stroke care in the months and years after stroke.

Our review has some important limitations. Many of the factors discussed here—such as secondary prevention, rehabilitation, and social reintegration strategies—have not been systematically examined in the EVT or LVO population. In the absence of better data, it is reasonable to extrapolate relevant insights from the general ischemic stroke population to help us optimize longer-term post-EVT care and outcomes in our current practice. Nevertheless, there remains a need for high-quality evidence from prospective cohort studies and longer-term follow-up of EVT trials or LVO cohorts to further validate the benefits of the various action items suggested in our paper. In addition, many of the insights about post-stroke care discussed in this paper have come from observational studies and are yet to be validated in randomized controlled trials. That being said, it is neither practical nor advisable to randomize patients into control arms for several non-pharmacological aspects like physician follow-up or societal accommodations for disability, so it is likely that we will have to continue relying on best-available observational data in many such cases. It is also important to note that various aspects of post-stroke care may not be generalizable to different healthcare systems owing to differences in care delivery and available resources.

When treating individual patients, stroke teams may perceive a loss of control over the long-term outcome of the patients as more time elapses post-stroke. Indeed, in the longtime horizon from 3 months to 5 years, several factors at the medical, psychosocioeconomic, and larger societal–environmental levels could erode EVT benefits. However, several factors at each level can also be leveraged to preserve or magnify treatment benefits, with opportunities to share responsibility with widening circles of care around the patient, involving primary care physicians, family/social supports, and policymakers. The race from stroke onset to EVT is a sprint, but the maintenance of EVT benefits from 3 months to 5 years post-stroke is a marathon.

## Author Contributions

AG co-conceived the paper, performed literature review, interpreted results, and wrote the first draft of the manuscript. JO and MM co-conceived the paper, performed literature review, interpreted results, and revised the manuscript. WZ, YR, and CM interpreted results and critically revised the manuscript. MG co-conceived the paper, provided supervision, and critically revised the manuscript. All authors contributed to the article and approved the submitted version.

## Conflict of Interest

AG reports membership in editorial boards of Neurology, Stroke, and Neurology Clinical Practice, and Stroke; speaker honoraria from NHS Health Education England; consulting fees from MD Analytics, MyMedicalPanel, Adkins Research Group, and Genome BC; research support from The Rhodes Trust, Wellcome Trust, the University of Calgary, Alberta Innovates, the Canadian Cardiovascular Society, and the Canadian Institutes of Health Research; stock/stock options from SnapDx, TheRounds.com, and Advanced Health Analytics (AHA Health Ltd.); and has a provisional patent application (US 63/024,239) for a system to deliver remote ischemic conditioning or other cuff-based therapies. JO is supported by the Julia Bangerter Rhyner Foundation, University of Basel Research Foundation, and Freiwillige Akademische Gesellschaft Basel. MG reports consulting fees from Medtronic, Stryker, Microvention, and Mentice and has a patent for Systems of stroke diagnosis licensed to GE Healthcare. The remaining authors declare that the research was conducted in the absence of any commercial or financial relationships that could be construed as a potential conflict of interest.

## Publisher's Note

All claims expressed in this article are solely those of the authors and do not necessarily represent those of their affiliated organizations, or those of the publisher, the editors and the reviewers. Any product that may be evaluated in this article, or claim that may be made by its manufacturer, is not guaranteed or endorsed by the publisher.

## Abbreviations

EVT: endovascular treatment
LVO: large vessel occlusion
mRS: modified Rankin Scale
QALY: quality-adjusted life year.
