# Supplementary material for: From Three-Months to Five-Years: Sustaining Long-Term Benefits of Endovascular Therapy for Ischemic Stroke
Source: Front Neurol. 2021 Jul 26;12:713738. doi: 10.3389/fneur.2021.713738 (PMC8350336; doi:10.3389/fneur.2021.713738)
Supplement: Supplementary file 1 [file Table_1.DOCX]

**APPENDIX: SEARCH STRATEGY**

We searched the electronic databases Medline/PubMed and EMBASE using the search terms outlined for each section below. To be included in this paper, studies needed to: (1) involve patients with ischemic stroke and (2) examine a post-stroke outcome of interest beyond the 3-month period. We limited the search to studies of humans published in English. The literature search is up-to-date as of 31 July 2020. The flow diagram for our search is shown in **Figure I.**

**I. Maximizing 3-month outcomes with EVT**

stroke AND (disability OR Rankin Scale OR outcome) AND (thrombectomy OR endovascular OR EVT OR aspiration OR intra-arterial)

The search was repeated with the following additional AND modifiers:

- Stent-retrievers OR device OR design

- Training OR Technique

- Stroke Unit OR stroke system OR integrated system

- Neuroprotection

- Machine-learning OR artificial intelligence OR AI

N=21,628 results

**II. Relationship between 3-month and 5-year post-stroke outcomes**

stroke AND disability OR Rankin Scale in combination with the following additional terms using the AND modifier:

- Recovery OR Rehabilitation

- Mortality OR Death

- Economic$ OR Cost$ OR Institutionalization OR long-term care

- Dementia

- Quality of life OR Utility

To specifically identify literature related to the long-term outcomes after endovascular therapy (EVT), we also repeated this search adding the following terms using the AND modifier: “thrombectomy” OR “endovascular” OR “EVT” OR “aspiration” OR “intra-arterial”

N=962 results

**III. Medical care-related factors influencing long-term stroke recovery**

- ischemic stroke AND secondary prevention AND strategies

- ischemic stroke AND follow-up visits AND outcome

- ischemic stroke AND 3-month follow-up AND long-term outcome

- ischemic stroke AND physician follow-up AND long-term outcome

- ischemic stroke AND secondary prevention AND compliance

- ischemic stroke AND medication adherence

- ischemic stroke AND long-term complications

- ischemic stroke AND late complications

- long-term complications AND ischemic stroke AND management

- post-stroke complications AND long-term outcome AND management

- ischemic stroke AND long-term complications AND infection

- post stroke infection AND long-term outcome AND management

- post-stroke dysphagia AND long-term outcome

- post stroke depression

- stroke AND patient education

- stroke AND (family physician OR primary care physician) AND outcome

N=1,325 results

**IV. Psycho-social factors influencing long-term stroke recovery**

- stroke AND (outcome* OR participat*) AND socio*

- stroke AND (outcome* OR participat*) AND econom*

- socioeconomic status AND ischemic stroke AND outcome

- stroke AND (social support OR family support OR caregiver)

- stroke AND (social support OR family support OR caregiver) AND outcome

- return to work AND stroke

**V. Societal and environmental factors influencing long-term stroke recovery**

- stroke AND (outcome* OR participat*) AND (environment* OR societ*)

- stroke AND (outcome* OR participat*) AND transport*

- stroke AND (outcome* OR participat*) AND barrier*

- stroke AND (outcome* OR participat*) AND system factor*

- stroke AND recover* AND education

- stroke AND recover* AND insurance

- stroke AND recover* AND (area OR geography*)

- stroke AND recover* AND access*

- stroke AND return AND work

- stroke AND recover* AND technology

N=2,773 results (for IV and V combined)

**
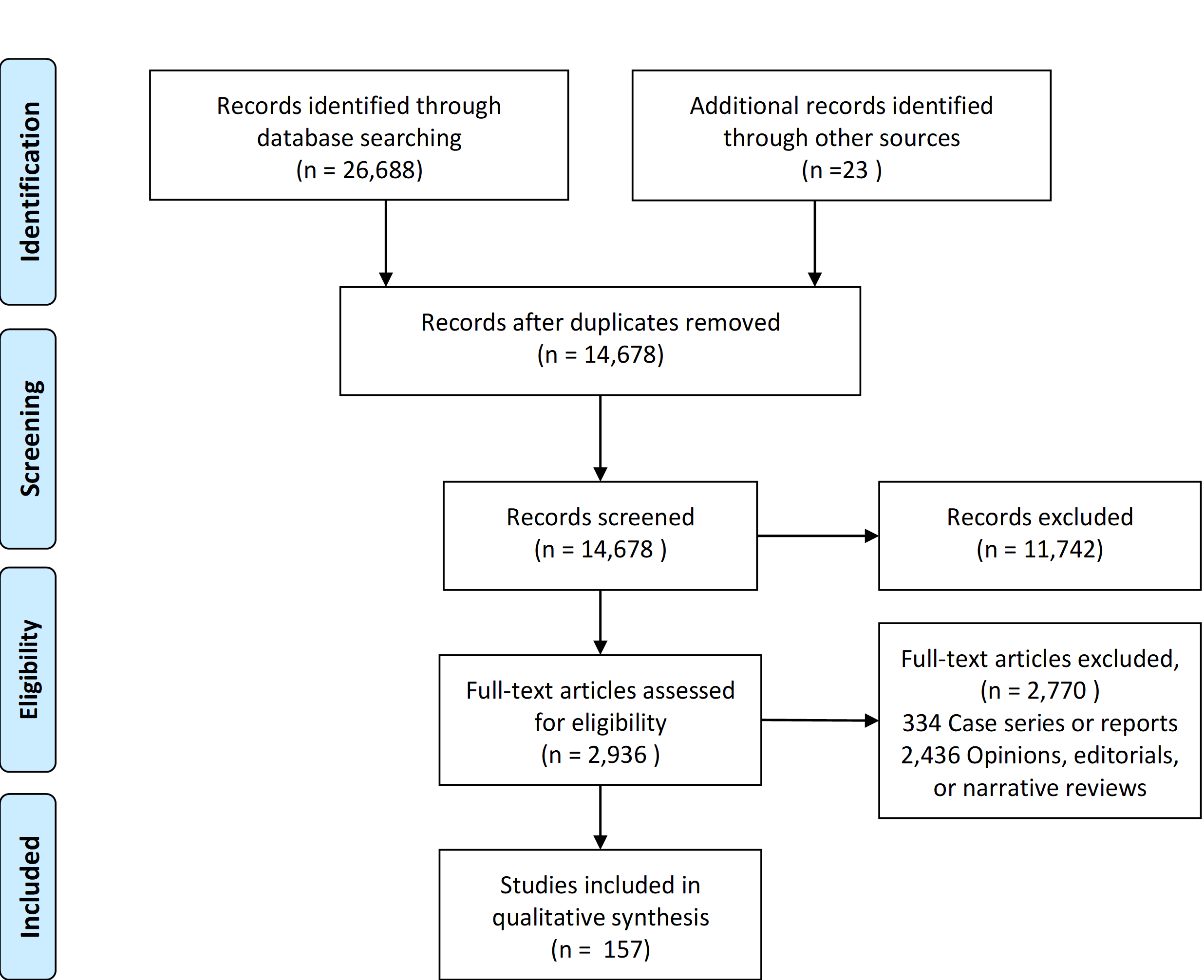
**

**Figure I.** PRISMA flow diagram for the studies included in our narrative review paper.
